# A Case of Apparently Threatened Phthisis Pulmonalis

**Published:** 1899-05

**Authors:** Frank W. Patch

**Affiliations:** South Framingham, Mass.


### A CASE OF APPARENTLY THREATENED PHTHISIS PULMONALIS.

Frank W. Patch, M. D., South Framingham, Mass.

Miss L. W., sixteen years of age, spare frame, dark complexion, weight 111½ pounds; both parents died of phthisis. Present condition in patient following la grippe. No distinct lesion in chest could be diagnosed by auscultation or percussion further than an abnormal respiratory rhythm; chest expansion very slight.

April 5th, 1895.—The symptoms presented were as follows: Dry, hollow cough throughout the day and until she sleeps at night, without expectoration; worse on first lying down; worse from laughing; chest feels "sore;" constipation; biting of nails; hands damp but warm; menses too profuse; very tired; appetite poor; gnawing in stomach after acids.

For the first two or three months there was little change. By careful attention to diet, chest exercises, and personal hygiene, her general health improved a little and she gained one or two pounds in weight, but the essential condition of illness remained unchanged by any of the five or six remedies already prescribed. The indications as above elicited were unsatisfactory and results correspondingly meagre.

June 24th.—A new picture was taken and the following additional symptoms were brought to view: Desire to be alone; cough always in paroxysms of two; salivation at night.

This cough was most singular, always deep, dry, and sepulchral in tone, two coughs at once with longer or shorter intervals (usually shorter) between the paroxysms. There was absolutely no expectoration. It was always possible to know of her approach toward my office on account of that unmistakable note of warning, which was easily recognizable anywhere within hearing distance. She now received a dose of Merc.-sol., CM (F.).

July 1st.—The day after taking the last remedy she began to have a steady, dull pain through temples; wakeful nights, followed by an attack of sick headache the next day with vomiting of yellowish matter; now dull, heavy feeling over whole head. I gave her S. L.

July 8th.—Severe backache; no loss of weight in past week. Merc.-sol. CM (F.).

July 20th.—Slightly less salivation; slight improvement in general feelings. Dr. Thurston saw the patient in consultation and made a careful physical examination; evidence negative, though we both thought the girl doomed to die of phthisis. The cough remains unchanged. Medorr. CM (F.).

July 27th.—No change. S. L.

August 3d.—Not as well. Merc.-v., 2C.

August 10th.—Slight improvement, no loss of weight; cough a little easier. S. L.

August 20th.—Cough improving. Two days before last menses had another severe sick headache lasting two days; better lying, worse from noise and light; better from hard pressure; vomiting of bile. (She had not previously been subject to sick headaches.) Merc.-v., 2C.

September 19th.—No headache at last menstrual period. I gave Merc.-v., 2C.

October 2d.—Cough improving; strength better; no further headache. Merc.-v., 2C.

November 4th.—Marked improvement, eats and enjoys three good meals daily; looks rosy and well; cough nearly gone; no further headache. S. L.

March 29th, 1897.—Has been very well up to the present month, when after a cold in the head the old cough has returned in the same paroxysms of two, hollow and barking; appetite failing; very slight salivation. A brother of the patient is slowly dying of consumption in an adjoining town, and the mental effect is evident here. Merc.-sol., CM (F.).

April 4th.—Very slight change. S. L.

April 12th.—Slight improvement. S. L.

May 4th.—Aching across the sacrum; appetite poor; feels weak and tired; cough not improving. Merc.-sol., 50M (F.).

May 22d.—Patient looks improved; backache better; good sleep; cough a little less deep and barking, but about the same in frequency; strength poor. S. L.

June 17th.—Slight improvement in some unessentials; cough same. Merc.-sol., 50M (F.).

July 24th.—Improvement. She picked twenty quarts of blackberries yesterday. S. L.

September 24th.—Has taken cold, which has brought an aggravation of the cough which had nearly left her. Merc.-sol., 50M (F.).

October 8th.—Still depressed from want of strength and "nervousness." Desire to be alone. Merc.-sol., 1M (F.).

October 19th.—Cough better; appetite and strength not improved. S. L.

November 9th.—Neuralgia in teeth and jaws for past two weeks; sharp, darting pain in sub-clavicular spaces. Bell., CM (F.).

November 23d.—Merc.-sol., 50M (F.). [The brother has just died; patient has been nursing him for several weeks.]

December 20th.—Much improvement, except for occasional attacks of neuralgia. S. L.

May 13, 1898.—Has not lost a day all winter from work in the shop, which involves a ride of eight miles in an open buggy. At present is suffering from attack of tonsilitis, right side; cough gone. Merc.-sol., 50M (F.).

In looking back over the experience of this case it is easy to point out some of the mistakes. The dose of Medorrhinum was one of these; there was no apparent excuse for it except in the sound of the cough and hope that it might serve to develop something on which to base a better prescription. It is also probable that there were too frequent repetitions of the Mercurius in the earlier part of the case, and possibly the 2C might have accomplished quicker results in the latter months than did the 50M. The lasting effect of the higher potencies ought, however, to turn the balance in their favor.

